# From a cell model to a fish trial: Immunomodulatory effects of heat-killed Lactiplantibacillus *plantarum* as a functional ingredient in aquafeeds for salmonids

**DOI:** 10.3389/fimmu.2023.1125702

**Published:** 2023-03-13

**Authors:** Sérgio Domingos Cardoso Rocha, Peng Lei, Byron Morales-Lange, Liv Torunn Mydland, Margareth Øverland

**Affiliations:** Department of Animal and Aquacultural Sciences, Faculty of Biosciences, Norwegian University of Life Sciences, Ås, Norway

**Keywords:** HK L-137, heat-killed lactobacillus plantarum L-137, paraprobiotics, RTgutGC, Atlantic salmon, immune-related biomarkers, microbiota, aquaculture

## Abstract

Paraprobiotics (dead/inactivated probiotics) are promising candidates in functional feeds to promote growth performance, modulate intestinal microbiota and enhance immune response of fish. During industrial production, fish are exposed to several stressful conditions such as handling, sub-optimal nutrition and diseases that can lead to reduced growth, increased mortalities and large economical losses. Such problems can be mitigated by use of functional feeds, leading to more-sustainable aquaculture and improved animal welfare. *Lactiplantibacillus plantarum* strain L-137 is a common bacterium found in fermented Southeast Asian dish made from fish and rice. The benefits of its heat-killed form (HK L-137) related to growth performance and immunomodulation have been studied in farmed fish such as Nile Tilapia (*Oreochromis niloticus*), striped catfish (*Pangasianodon hypophthalmus*) and bighead catfish *(Clarias macrocephalus*). To study if such benefits can also be observed in salmonids, we worked both at *in vitro* level using an intestinal epithelium cell line from rainbow trout (*Oncorhynchus mykiss*; RTgutGC) stimulated with HK L-137 (Feed LP20™) and at *in vivo* level with pre-smolt Atlantic salmon (*Salmo salar*) fed HK L-137 at different inclusion levels (20, 100 and 500 mg of Feed LP20™ kg^-1^ feed). In RTgutGC, the results showed that the barrier function of the cell monolayer was strengthened along with an increased production of IL-1β and a decreased production of Anxa1, indicating a modulation of the immune response. Interestingly, a similar trend was detected at the *in vivo* level in distal intestine from fish fed the highest inclusion level of HK L-137. Here, a lower production of Anxa1 was also detected (after a 61-day feeding period) in addition to an increase of total plasma IgM in the same group. Furthermore, the RNA-seq analysis showed that HK L-137 was able to modulate the gene expression of pathways related to molecular function, biological process and cellular component in distal intestine, without compromising fish performance and gut microbiota. Taken together, our study has shown that HK L-137 can modulate physiological response of Atlantic salmon, making fish more robust against stressful conditions during production.

## 1 Introduction

The rapid development of aquaculture in recent decades can significantly contribute to meet global demand for animal-derived protein (1). However, the aquaculture industry faces considerable economic losses during the grow-out period due to smolts of low quality combined with exposure to multi-stressor conditions such as stress due to handling, sea water transportation, delousing, poor water quality and exposure to pathogens, leading to diseases and high mortality (2). A cost-effective way to prevent such losses is the use of functional feeds with immunomodulatory properties during freshwater phase to promote a more resilient fish. The intestine is an interesting target for this type of approach, since the mucosa-associated lymphoid tissue (MALT) in this organ (GALT) can coordinate both local and systemic immune responses (e.g., inflammation, antigen presentation process, T cell polarization, production of effector molecules and cytokines), which modulate the fish’s response to a challenge (3–5). In addition, intestinal microbiota, due to its modulatory capacity, could be the link between diet, immune system and host health (6).

*Lactiplantibacillus plantarum* (previously known as *Lactobacillus plantarum*), specifically the strain L-137, is found in fermented Southeast Asian dish made from fish and rice (7, 8). This Gram-positive bacterium is a lactic acid producing bacteria that contains lipoteichoic acid on its cell wall surface which can be recognized by pattern recognition receptors in the host cells (9–11). Human trials have shown an array of benefits, such as reduced incidence of upper respiratory tract infections, improved immune function, lipid metabolism, intestinal barrier function and general quality of life (12–15). Moreover, *in vitro* and *in vivo* studies in mice demonstrated that the heat-killed form of this strain (HK L-137) induces regulatory cytokines such as IFN-γ and IL-12

Functional feeds containing prebiotics and/or probiotics have great potential to improve growth performance and immunity along with fish health and welfare (16–19). Consequently, they can contribute to reduce mortality and increase sustainability of fish production. Paraprobiotics (dead/inactive probiotics) have also shown to be beneficial (20, 21). Because the bacteria is inactivated, feed storage is easier and the impact on the intestinal microbiota is more predictable compared to feed containing probiotics, as bacteria is unable to grow and dominate the microbial community (21). HK L-137 has a large potential in functional feeds. Its use has been associated with increased growth performance or immunomodulation in aquaculture species like giant freshwater prawn (*Macrobrachium rosenbergii*) (22), sea cucumber (*Apostichopus japonicus*) (23), red sea bream (*Pagrus major*) (24), Nile tilapia (*Oreochromis niloticus*) (25), striped catfish (*Pangasianodon hypophthalmus*) (26) and bighead catfish (*Clarias macrocephalus*) (27).

Bioactive properties of HK L-137 has been reported to be dose-dependent and species-specific (20, 22–27). Considering this, *in vitro* cell-based models are useful tools to study the immunomodulatory properties of a novel feed component. Such models can reduce the number of animal studies, have higher throughput and lower cost, can allow for adjustments and optimization of inclusion levels, exposure duration and as well as to assist in determining target tissues to sample before larger *in vivo* experiments.

In the present study, we aimed to evaluate the effect of paraprobiotic HK L-137 as a bioactive component in functional feed for Atlantic salmon. To study the possible immunomodulating effects of HK L-137, we performed an *in vitro* trial using a rainbow trout-derived intestinal epithelium cell line (RTgutGC) exposed to different concentrations of pre-digested HK L-137 to mimic *in vivo* gastro-intestinal digestion. Following the *in vitro* trial, an *in vivo* salmon feeding trial was performed with increasing doses of HK L-137. At both the *in vitro* and the *in vivo* level, HK L-137 was shown to act as an immunostimulant by modulating cytokines and effector molecules, suggesting a potential in functional feeds for salmonids, without compromising fish performance and gut microbiota. Further, the results demonstrated the benefits of performing *in vitro* studies first, to reduce animal experiments and improve animal welfare, when studying novel ingredients and additives. This approach creates a baseline for future work involving HK L-137 and other pro- and paraprobiotics, both *in vitro* and *in vivo*.

## 2 Materials and methods

### 2.1 Heat killed *L. plantarum* strain 137

The heat-killed strain of *L. plantarum* (HK L-137) was produced and provided by House Wellness Foods Corporation (Itami, Japan) in the commercial name of Feed LP20™ (20% HK L-137 and 80% tapioca dextrin in dried-weight basis). *L. plantarum* was heat-killed at 70° C for 10 minutes, with a final concentration in the dry product of 2×10^11^ CFU g^-1^ (8).

### 2.2 *In vitro* assays

As *in vitro* model, RTgutGC cell line derived from rainbow trout (*Oncorhynchus mykiss*) intestinal cells was used. Routine RTgutGC cell culture was based on the description by Kawano et al. (28) and detailed information about their maintenance is presented in Wang et al. (29).

#### 2.2.1 *In vitro* pre-digestion of HK L-137

To mimic the *in vivo* gastro-intestinal digestion, Feed LP20™ was pre-digested before exposing to cells in a two-step *in vitro* system. To 2 g of Feed LP20™, 9.6 mL pepsin-solution (pepsin 416.7 U mL^-1^ in 0.084 mM HCl, 35 mM NaCl, pH 2.0) and 0.4 mL chloramphenicol-solution (0.1% w v^-1^) were added in a 50 mL screw cap tube. The solution was incubated in a shaking water bath at 37°C for 2 hours. After incubation, 675 µL 1 M NaOH was added to inactivate pepsin activity and pH was adjusted to 7.8 by adding 10 mM PBS. The solution went through another incubation in a shaking water bath at 37°C for 1 hour to stabilize the temperature again. Later, 0.6 mL of an intestinal enzyme cocktail (trypsin 2100 U mL^-1^, chymotrypsin 100 U mL^-1^ and elastase 0.2 U mL^-1^ in 10 mM PBS pH 7.8), was added and incubated in the shaking water bath at 37°C for 6 hours. After digestion, the solution was immediately kept in boiling water for 5 minutes to deactivate the enzymes and total solution was freeze-dried. A stock solution was made with a resuspension in PBS at 20 mg mL^-1^ for later *in vitro* assays. All compounds and enzymes were supplied from Sigma-Aldrich, Germany.

#### 2.2.2 Assessment of cell viability

RTgutGC cells were exposed to a serial dilution of HK L-137. Briefly, in a 96-well plate (CLS3340-50EA, Merk), 1.5×10^5^ cells mL^-1^ were seeded out together with L15 culture medium (100 µL per well). When 85-90% confluency was achieved, four concentrations of HK L-137 (0, 1, 10, 20 and 40 µg mL^-1^) were dissolved in L15 medium and added to the cells. After 24 hours induction, the cells were washed with PBS twice, and 10 µL of AlamarBlue (DAL1025, Thermo Fisher) in 100 µL of L15 medium was added to the wells and incubated for 2 hours in dark at room temperature. Then, fluorescence signal at 540/610 nm was measured by SpectraMax microplate reader (Molecular Devices). The assay was performed three times with three technical replicates.

#### 2.2.3 TEER and permeability of the monolayer

As a quality measure of monolayer formation, transepithelial electrical resistance (TEER) was detected in RTgutGC cells grown on permeable membrane for 31 days (29, 30). Cells were exposed to 40 µg mL^-1^ of HK L-137 and TEER was measured after 6 and 24 hours by using an EVOM Volt Ohm Meter with STX1 electrode (World Precision Instruments, Germany). Thereafter, cells were washed with PBS twice and 30 µM of Lucifer yellow (L1177, Thermo Fisher) was added to the culture medium in the apical side of the membrane, followed by a two-hour incubation at 20°C. Lastly, 100 µL of each sample was collected from basolateral chamber and the fluorometric signal was read at 428/536 nm using SpectraMax microplate reader (Molecular Devices). The assay was performed three times with three technical replicates.

#### 2.2.4 Indirect ELISA

To detect protein production of specific biomarkers in RTgutGC cells related with HK L-137 exposure, cells were seeded at 1×10^6^ cells per well (6-well plates). When the cells reached 90% confluency, culture media were exchanged with 40 µg mL^-1^ of HK L-137 for 6 and 24 hours. Pro-inflammatory cytokine interleukin 1 beta (IL-1β), anti-inflammatory protein Annexin a1 (Anxa1) and tight junction protein E-cadherin (E-cad) were assessed through indirect ELISA. Total proteins of each sample (in RIPA lysis buffer: 89901, Thermo Fisher) were quantified using the BCA Protein Assay Kit (23225, Thermo Fisher) following the manufacturer’s instructions. Then, as described previously (31), each sample was diluted in bicarbonate buffer (60 mM NaHCO_3_ pH 9.6) and seeded in duplicate in a 96-well plate (Nunc, Thermo Fisher) at 50 ng µL^-1^ (100 µL) for overnight incubation at 4°C. Next, 200 µL of blocking solution (37587, Thermo Fisher) was incubated per well for 2 h at 37°C. Later, the plates were incubated for 90 min at 37°C with 100 µL of the first antibody (**Table 1**) and for 60 min at 37°C with a secondary antibody (100 µL) diluted 1:5000 (goat anti-mouse IgG-HRP, 31430, Thermo Fisher or mouse anti-rabbit IgG-HRP, sc-2357, Santa Cruz Biotechnology). The assay was performed three times with two technical replicates.

**Table 1:** Primary antibodies for indirect ELISA.

Marker	Source	Type	Dilution	Reference
IL-1β	Rabbit	Polyclonal	1:1000	Boltaña et al., 2018 (32)
Anxa1	Mouse	Polyclonal	1:500	**Supplementary Figure 3**
E-cad	Rabbit	Monoclonal	1:500	701134, ThermoFisher
IgM	Mouse	Monoclonal	1:500	FM-190AZ-5, Ango

IL-1β, Interleukin 1; beta, Anxa1, Annexin a1; E-cad, E-cadherin; IgM, Immunoglobulin M.

### 2.3 *In vivo* experiment

#### 2.3.1 Experimental diets

Five experimental diets were produced at NMBU Center for Feed Technology (Fôrtek, Ås, Norway). The diets consisted of a fish meal and plant-based commercial-like diet, without any growth-promoting feed additives. HK L-137 was supplied in the diets in four concentrations: 0 (control diet), 20, 100 and 500 mg of Feed LP20™ kg^-1^ feed (CD, LP20, LP100 and LP500, respectively). Moreover, a positive control diet (MG) with the addition of 2 g kg^-1^ of β-glucan (MacroGard^®^, Biorigin, Brazil) was also produced.

The diets were formulated to meet the nutrient requirements for high-performing pre-smolt under commercial conditions (33). The feed formulation is presented in **Tables 2**, **3**, respectively. Briefly, dry ingredients were thoroughly mixed in a professional small-scale commercial paddle mixer for four minutes. The mix was divided in five batches and then added the intended amounts of Feed LP20™ or β-glucan, and then the liquid ingredients. The mash was cold-pelleted into 2.5 mm of diameter by using P35A pasta extruder (Italgi, Italy). The pellets were dried in small experimental dryers at approximately 60°C until 8-10% humidity and stored at 4°C prior to feeding. Chemical analysis of the diet composition (ground at 0.5mm) was performed in duplicates by the LabTek group at the Department of Animal and Aquacultural Sciences, NMBU (**Table 3**). Dry matter, ash, crude protein, crude lipid, and amino acids were analyzed according to the methods described in the European Commission Regulation No 152/2009 (34). Starch was hydrolyzed with α-amylase and amyl glucosidase-enzymes to glucose, and the glucose concentration was determined spectrophotometrically (RX4041 Randox Daytona+, Randox Laboratories, Antrim, UK) as described by McCleary et al. (35). Gross energy content was determined using a PARR 6400 Automatic Isoperibol Calorimeter (Parr Instruments, Moline, Illinois, USA) according to ISO 9831 (36). Total P and Y contents were measured using a microwave plasma atomic emission spectrometer (MP-AES 4200, Agilent Technologies, USA) after combustion and acid decomposition in a Start D microwave digestion system (Milestone Srl, Italy).

**Table 2:** Diet formulation of experimental diets.

Ingredients	(g kg^-1^)
Fishmeal^a^	278.7
Soy protein concentrate^b^	177.0
Wheat gluten^c^	65.6
Pre-gelatinized potato starch^d^	98.4
Gelatin^e^	98.4
Fish Oil^f^	262.3
Monocalcium phosphate^g^	12.3
L-lysine^h^	1.2
DL-Methionine^i^	0.8
Choline chloride^j^	1.2
Vitamin/Mineral premix^k^	4.1
Yttrium oxide^m^	0.08

^a^LT fishmeal, Norsildmel, Egersund, Norway; ^b^Soybean meal, Denofa AS, Fredrikstad, Norway; ^c^Wheat gluten, Amilina AB, Panevezys, Lithuania; ^d^Lygel F 60, Lyckeby Culinar, Fjälkinge, Sweden; ^e^Rousselot 250 PS, Rousselot SAS, Courbevoie, France; ^f^NorSalmOil, Norsildmel, Egersund, Norway; ^g^Monocalcium phosphate, Bolifor MCP-F, Oslo, Norway Yara; ^h^L-Lysine CJ Biotech CO., Shenyang, China; ^i^Rhodimet NP99, Adisseo ASA, Antony, France; ^j^Choline chloride, 70% Vegetable, Indukern SA., Spain; ^k^Vit/min premix, NMBU Fish Premix Basic, Trouw Nutrition, LA Putten, The Netherlands. Per kg of feed: Vitamin A 2500 IU; Vitamin D2 1500 IU; Vitamin E (all-rac-alpha-tocopheryl acetate) 200 IU; Vitamin K3 (Menadione nicotinamide bisulfite) 10.05 mg; Vitamin B1 (Thiamine mononitrate) 15.0 mg; Vitamin B2 (Riboflavin) 25.0 mg; Calcium D-pantothenate 40.0 mg; Niacinamide 75.05 mg; Vitamin B6 (pyridoxine hydrochloride) 15.0 mg; Folic acid 5.0 mg; Vitamin B12 (cyanocobalamin) 25.0 mg; Vitamin C 125.0 mg; Biotin 275.0 mg; Calcium iodate, anhydrous, Iodine 30.0 mg; Manganese (II) oxide, Manganese 15.0 mg; Zinc oxide, Zinc 105.0 mg. Carrier: Calcium carbonate; ^m^Ytrium Oxide (Y_2_O_3_), Metal Rare earth Limited, Shenzhen, China.

**Table 3:** Analyzed chemical composition.

	CD	LP20	LP100	LP500	MG
Dry matter (%)	93.3	91.9	92.3	93.2	93.4
Crude protein (%)	45.7	45.0	45.1	45.1	45.3
Crude lipid (%)	23.8	24.3	21.6	21.7	24.1
Ash (%)	6.70	6.53	6.65	6.54	6.59
Starch (%)	10.9	10.3	10.3	10.5	10.5
Energy (MJ kg^-1^)	23.4	23.2	23.2	23.5	23.4
Total phosphorous (g kg^-1^)	11.7	11.4	11.4	11.4	11.5
Amino acids (g kg^-1^)					
Alanine	19.11	18.98	18.81	18.09	18.67
Arginine	24.80	24.85	25.28	23.85	24.83
Aspartic acid	36.04	33.35	33.97	33.60	34.40
Cysteine	4.00	3.82	3.99	3.78	3.86
Glutamic acid	77.61	76.29	77.16	75.91	77.73
Glycine	24.61	24.48	24.62	24.37	25.01
Histidine	9.77	9.07	8.93	9.13	9.08
Isoleucine	14.80	14.44	14.59	14.47	15.00
Leucine	26.36	25.76	25.94	25.04	25.77
Lysine	26.31	24.91	25.10	25.13	25.57
Methionine	8.17	7.70	7.74	7.26	8.02
Phenylalanine	17.27	16.59	16.56	15.03	16.31
Proline	25.46	24.64	25.12	25.56	22.72
Serine	17.33	16.22	16.20	15.51	16.36
Threonine	15.26	13.79	14.08	13.60	14.97
Tyrosine	9.60	10.37	10.44	10.37	10.74
Valine	14.02	13.62	13.49	13.64	13.76
Sum amino acids (g kg^-1^)	370.5	358.9	362.0	354.3	362.8

CD, control diet; LP20, LP100 and LP500, 20, 100 and 500 mg of Feed LP20™ kg^-1^; MG, positive control diet.

#### 2.3.2 Experimental design

The fish trial was conducted at the Center of Sustainable Aquaculture at the Norwegian University of Life Science (NMBU), Ås, Norway. The experimental procedures were performed in accordance with the national guidelines for the care and use of animals (The Norwegian Animal Welfare Act and the Norwegian Regulation and Animal Experimentation). A total of 825 Atlantic salmon (AquaGen Atlantic QLT-innOva SHIELD) were randomly distributed into 15 fiberglass tanks (300 L) with average biomass per tank of 1500 ± 2.7 g (55 fish per tank with average individual weight of 27.3 g). Fish were kept under continuous light and recirculated fresh water with a water supply of 8 L min^-1^ and average water temperature of 14.9°C. Dissolved oxygen levels were kept above 7.0 mg L^-1^ in the water outlet. Each experimental diet was supplied in triplicate tanks over a period of 61 days. Fish were fed *ad libitum* with 10% excess by electrically driven belt feeders once a day for 6h. The amount of feed in the feeders were adjusted daily based on the expected fish biomass in the tanks and the uneaten feed in each tank. Uneaten pellets were collected within one hour after feeding from the outlet water settling on a screen for each tank, according to a method described previously (37). Feed intake, biomass gain, feed conversion ratio and specific growth rate were calculated as described previously (38).

#### 2.3.3 Final sampling

At the end of the experimental period, 6 fish per tank were sampled at once, anesthetized by using tricaine methane sulfonate (MS-222, 50 mg L^−1^ water) and killed by a sharp blow to the head. The individual weight of sampled fish was registered and was added to the pooled weight of the remaining fish from each tank. Blood was collected with heparinized syringe from the dorsal vein. After centrifugation (3,000 x g for 10 min at 4°C), plasma was collected and stored at -80°C until further analysis.

Each fish was opened with a sterile scalpel, the intestine was removed and cleared of any mesenteric and adipose tissue. Then, distal intestine was open longitudinally. Digesta was scraped gently with plastic spatula into cryotubes, snap frozen in liquid nitrogen and stored at -80°C until further analysis. A proximal section of the distal intestine was rolled and fixed in 10% formalin (for histology analysis) and distal section was preserved in RNAlater, stored at 4°C for 24h and finally at -80°C until analysis.

#### 2.3.4 Detection of protein biomarkers

To detect immunological markers in distal intestine (IL-1β, Anxa1 and E-cad) and total IgM in plasma samples (n=9 per dietary group), an indirect ELISA was carried out, after quantifying the total proteins of each sample by the BCA method. Furthermore, for the detection of plasma specific IgM (39) against Feed LP20™, 50 ng µL^-1^ total protein extract of Feed LP20™ were seeded by duplicate in a 96-well plate. After overnight incubation and blocking, each fish plasma was incubated in duplicate at 70 ng µL^−1^ total IgM (100 µL) for 90 min at 37°C. Then, the ELISA protocol described above (section 2.2.4) was followed.

#### 2.3.5 Histological examination of distal intestine

Formalin-fixed distal intestine samples were treated with standard histological techniques (ethanol-xylene-paraffin embedding). Longitudinal sections of 6 µm in thickness were prepared. The sections were stained with hematoxylin and eosin. Images were captured using a DMLS light microscope (Leica Microsystems, Wetzlar, Germany) equipped with a Leica E3 digital imaging camera and LAS EZ v4.9 software. The measurement of 6-10 villi height per fish was done from the stratum compactum to the tip of the single fold by ImageJ software. Only finger-like single folds were measured and only one single fold per complex folds inter-space. Images were analyzed blindly and samples with poor quality or insufficient number of finger-like single folds were excluded.

#### 2.3.6 Extraction of DNA from samples

To evaluate the effect of the HK L-137 on intestinal microbiota, gene-based sequencing approach of the V3–V4 hypervariable regions of the bacterial 16S rRNA gene was performed. The total DNA of all digesta samples was extracted using QIAamp Fast DNA Stool Mini Kit (Qiagen, cat. #51604) according to the guidelines of the manufacturer as described previously (40). DNA from feed (with technical duplicate) and water from all the tanks was also extracted with the same methodology (40). To ensure the reliability of the present workflow, blank samples were included during both extraction and sequencing, as well as DNA from a positive control with a known microbial community (catalog no. D6300, Zymo- BIOMICS™). After purification of the PCR products with Agencourt AmPure XP beads (Beckman Coulter) and examination by 1% agarose gel electrophoresis, the 12 digesta samples with the most defined and strong bands within each dietary group were used for sequencing.

#### 2.3.7 Library preparation and 16S sequencing processing

The library preparation was performed according to the Illumina 16S Metagenomic Sequencing Library Preparation protocol. Library was loaded at 8 pM and sequenced on the Miseq System using the Miseq Reagent Kit v3, 600-cycle (Illumina). Further information and scripts are described elsewhere (40). Briefly, the analysis of sequenced data was done in R 4.1.0 (41) with DADA2 1.18.0 package (42) to generate amplicon sequence variants (ASVs) with 22nt overlap. The resulting ASVs were assigned with taxonomy reference database SILVA (v138.1) (43, 44). ASVs identified as chloroplast, mitochondria, cross-contamination and no phylum-level taxonomic assignment were removed. After clustering by using VSEARCH algorithm and curated with LULU (45), 1593 unique ASVs and 515 identified taxonomic groups were identified, from which, 71.1% at the genus level. Alpha- and beta-diversities were calculated according with previous literature (39, 40).

#### 2.3.8 Transcriptomics in distal intestine

Total RNA was extracted from 45 distal intestine samples (three per dietary group from triplicate tanks), using Qiazol Lysis Reagent (Qiagen, cat. #79306) recommended protocol, based on chloroform and isopropanol standard method. RNA was eluted in 30 µL and concentration and RIN was measured through Nanodrop (Nanodrop Technologies) and BioAnalyzer (Agilent), respectively. All samples satisfied the desired quality parameters, including RIN ≥ 8. Library preparation and RNA-seq were performed by NovoGene (Cambridge, UK), by using paired-end 150 bp sequencing strategy (Illumina NovaSeq platforms), with an average target of 26.6 million reads/sample.

### 2.4 Statistical analysis

Data analyses (means, standard deviation and multiple comparisons test) and graphical presentation of the results were done using GraphPad Prism 8.0.2. One-way ANOVA analysis was followed by pairwise comparison using Tukey’s test for multiple comparisons. When not following normal distribution (Shapiro-Wilk test), non-parametric Mann-Whitney test was used and when necessary, Student’s t-test (two-tailed) was used to determine significant differences between the different experimental diets and the control diet. All differences were considered significant at p < 0.05.

RNA-seq raw data analysis was performed as recently reported (46), by using nf-core rnaseq v3.3 (47) in Orion (NMBU- High-Performance Computing cluster). Cleaned reads were aligned to *Salmo salar* genome SSAL_v3.1 (GenBank assembly accession: GCA_905237065.2) and fragments mapping were counted using featureCounts (subread v1.5.1). Differentially expressed genes (DEGs) were estimated using SARTools R package (v1.7.3) when adjusted p-value (padj) was < 0.05. ShinyGO v0.76.3 (48) was used to perform the enrichment analysis and functional classification of DEGs (by KEGG database). Term categories (FDR < 0.05) were displayed and sorted by Fold Enrichment (minGSSize = 4).

## 3 Results

### 3.1 *In vitro* trial

**Figure 1:**
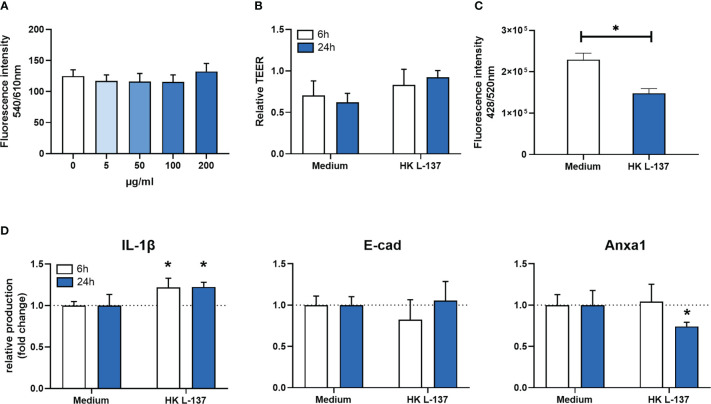
*In vitro* results with RTgutGC cell line after HK L-137 exposure. **(A)** cell viability after 24 hours of exposure to Feed LP20™ at differences concentrations. Data is presented as mean (SD) per dosage; **(B)** Relative transepithelial electrical resistance (TEER) in monolayer of RTgutGC cells with and without exposure to Feed LP20™ for 6 and 24 hours. Data is presented as mean (SD) per treatment and compared between time points. **(C)** Lucifer yellow permeability in monolayer of RTgutGC cells with and without exposure to Feed LP20™ for 6 hours. Data is presented as mean (SD) per treatment; **(D)** Indirect ELISA results from RTgutGC cell line after 6- and 24-hour treatment. Data is represented in fold change (n=3); **(A–D)** error bars represent the standard deviation (n=3). Asterisk (*) denote level of significance (*p-value < 0.05). IL-1β: Interleukin 1β; E-cad: E-cadherin; Anxa1: Annexin A1.

After exposing RTgutGC cells to different doses of Feed LP20™ for 24 hours, we observed no difference in cell viability (**Figure 1A**), even for the higher exposure levels. RTgutGC monolayer showed no changes in the transepithelial electrical resistance (TEER) after 6- and 24-hours exposure to HK L-137 (**Figure 1B**). Nevertheless, a significantly decreased (p-value = 0.002) paracellular permeability was detected after 6-hours exposure to HK L-137 (**Figure 1C**).

The detection of immune biomarkers (**Figure 1D**) showed that IL-1β production by RTgutGC was higher in cells exposed to HK L-137 after 6 (p-value = 0.011) and 24 hours (p-value = 0.021). At the same time, Anxa1 production was significantly lower, but only at the 24-hour time point (p-value = 0.031). Production of E-cad did not change significantly after HK L-137 exposure at any time point.

### 3.2 *In vivo* experiment

#### 3.2.1 Fish performance

No significant difference was found between dietary groups regarding feed intake, body weight gain, specific growth rate and feed conversion ratio, as presented in **Table 4**.

**Table 4:** Performance parameters.

	CD	LP20		LP100		LP500		MG	
Feed intake (g DM fish^-1^)	47.54 (± 2.80)	53.53 (± 5.76)		47.96 (± 5.79)		45.86 (± 1.52)		53.29 (± 4.32)	
Body weight gain (g fish^-1^)	57.66 (± 3.94)	>60.74 (± 2.31)		57.55 (± 6.87)		55.14 (± 3.24)		64.35 (± 5.61)	
Specific growth rate (%BW day^-1^)	1.802 (± 0.075)	1.861 (± 0.041)		1.797 (± 0.124)		1.753 (± 0.061)		1.921 (± 0.096)	
Feed conversion ratio	0.825 (± 0.012)	0.880 (± 0.063)		0.833 (± 0.014)		0.833 (± 0.022)		0.829 (± 0.017)

Results are presented as mean (± SEM). DM, dry matter; CD, control diet; LP20, LP100 and LP500, 20, 100 and 500 mg of Feed LP20™ kg^-1^; MG, positive control diet.

#### 3.2.2 Intestinal microbiota

**Figure 2:**
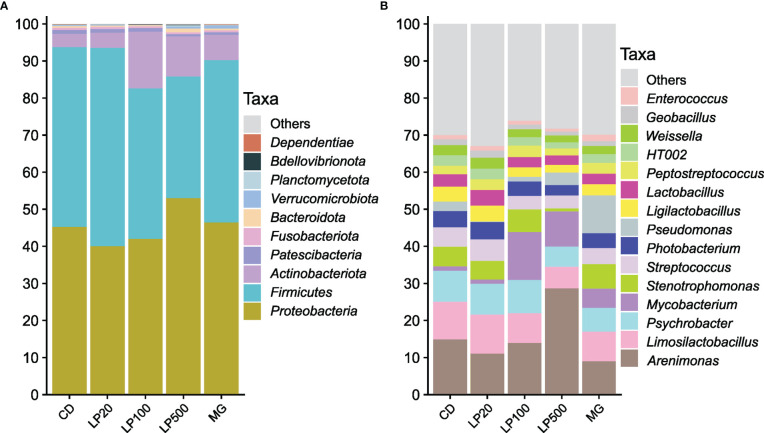
Relative abundance of the most abundant taxa in distal intestine digesta samples from fish fed experimental diets. Top 10 most abundant taxa at phylum **(A)** and top 15 most abundant taxa at genus or lower taxonomy level **(B)**; CD, control diet; LP20, LP100 and LP500: 20, 100 and 500 mg of Feed LP20™ kg^-1^, respectively; MG: positive control diet.

Regarding digesta samples, more than 95% of all bacteria in digesta belong to the phyla *Proteobacteria*, *Firmicutes* and *Actinobacteriota* (**Figure 2A**). *Proteobacteria* and *Firmicutes* relative abundances did not differ from the control group, while *Actinobacteriota* abundance was significantly increased by the highest inclusion levels of HK L-137 (p-value < 0.0001 and p-value = 0.021, respectively, **Figure S1**). At lower taxonomic level, *Arenimonas, Limosilactobacillus, Psychrobacter* and *Mycobacterium* were the most abundant genera (**Figures 2B**, **S1**). Relative abundance of *Mycobacterium* was significantly different from the CD group (except in LP20 group), while higher inclusion of HK L-137 led to a lower relative abundance of the genera *Stenotrophomonas* and *Ligilactobacillus* (**Figure S1**).

With a prevalence threshold of 80%, 53 ASVs were identified as core microbiota, with 28 of those shared by all experimental groups. Three ASVs, two classifieds as *Limosilactobacillus* and one as *HT002*, were identified as core ASVs in all the digesta samples.

**Figure 3:**
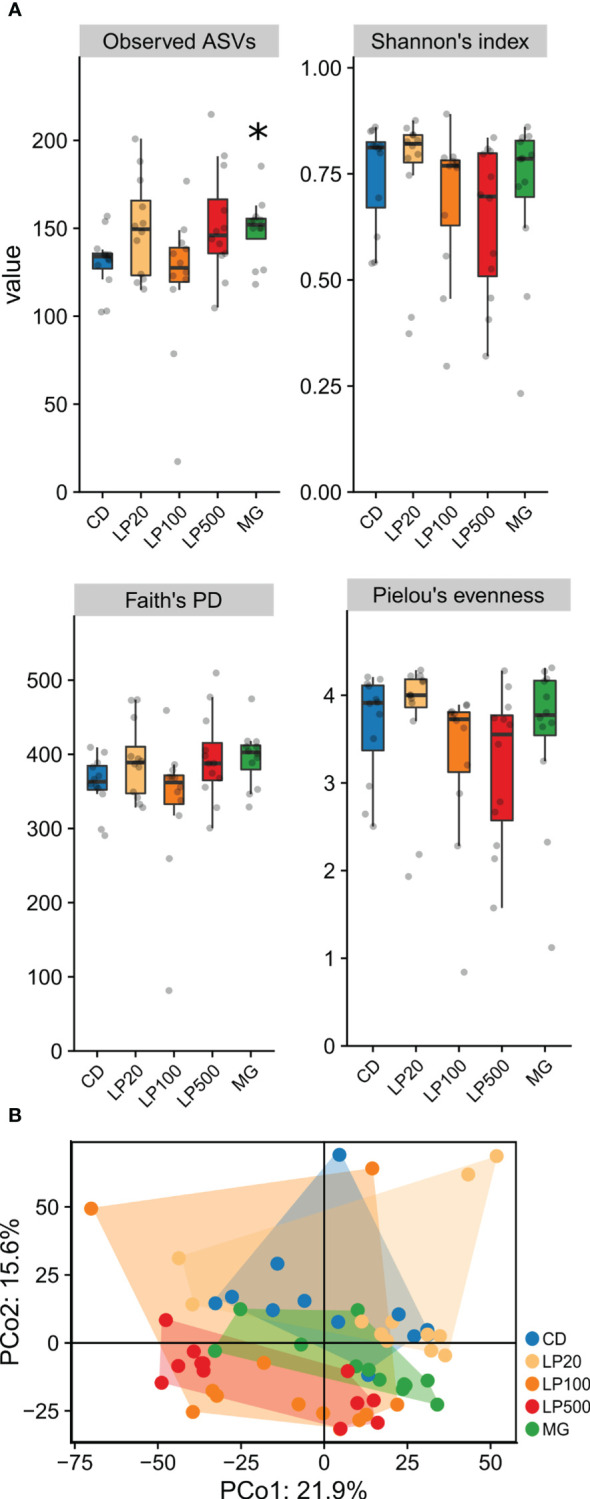
Microbial diversities of gut microbiota. **(A)** alpha-diversity represented in fours indices. Asterisk (*) denotate statistically significant differences when compared with control diet (*p* < 0.05) after Wilcox pairwise comparison (n=12). **(B)** beta-diversity represented as PCoA of PhILR transformed data (n=12). ASVs, amplicon sequence variants; PD, phylogenetic diversity; PCo, Principal component; CD, control diet; LP20, LP100 and LP500: 20, 100 and 500 mg of Feed LP20™ kg^-1^, respectively; MG, positive control diet.

When studying microbial diversity, alpha-diversity analysis (**Figure 3A**) showed that the control diet group (CD) was only significantly different from the positive control (MG) at the observed number AVSs (p-value = 0.015), indicating increased richness. Other indices, such as Shannon’s index (comprehending richness and evenness), Pielou’s evenness index and even Faith phylogenetic diversity index, had no significant differences between treatment groups and the control diet. Regarding beta-diversity, between-sample diversity, principal coordinate analyses (PCoA) of Phylogenetic Isometric Log-Ratio Transform (PhILR) transformed data with centroid distance explains 21.9% and 15.6% of the data. There was a considerable dispersion and the group with higher inclusion level, LP500, was the only one showing no overlap with the CD group, with the MG control group in between them (**Figure 3B**). Permutational multivariate analysis of variance (PERMANOVA) test indicates that there were differences between groups (p-value = 0.001), however, a pairwise comparison post-test only identified differences between the positive control (MG) and the remaining diets.

### 3.3 Histology

Histological analyses showed no significant difference in single folds height between groups (**Figure S4**) or any signs of inflammation in the distal intestinal samples.

### 3.4 Transcriptomics

The transcriptomic analysis showed that increasing inclusion level of HK L-137 in the diets induced an increase in differentially expressed genes in the distal intestine. The comparison between experimental diets and control is summarized in **Table 5**.

**Table 5:** Significant differentially expressed genes (DEGs) and Term Enrichment per comparison.

	DEGs		Term Enrichment	
Comparison	Up-regulated	Down-regulated	Up-regulated	Down-regulated
LP20 | CD	647	159	5	2
LP100 | CD	2692	888	13	9
LP500 | CD	2694	1034	15	8
MG | CD	2	0	0	0

CD, control diet; LP20, LP100 and LP500, 20, 100 and 500 mg of Feed LP20™ kg^-1^; MG, positive control diet.

**Figure 4:**
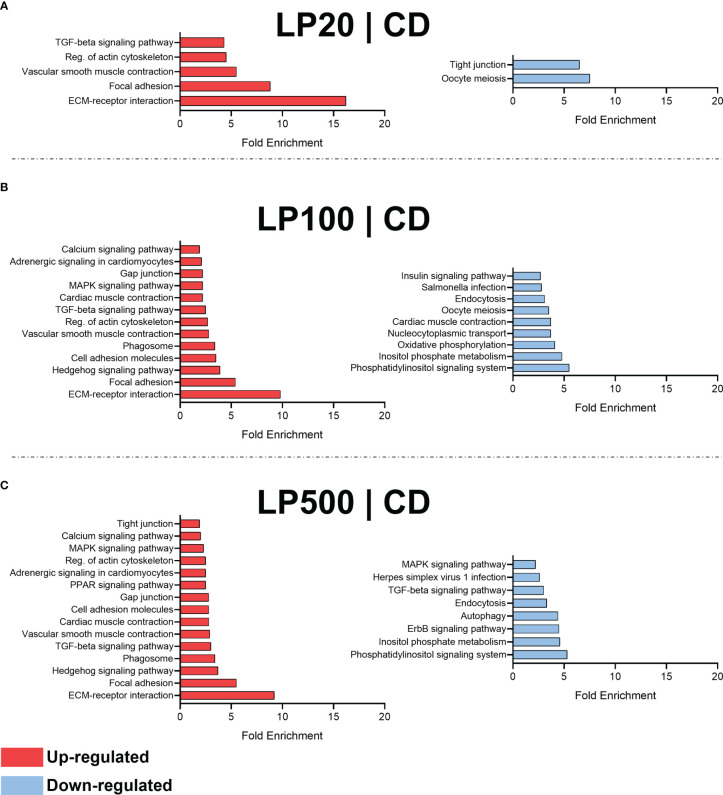
Enriched terms by ShinyGO. Comparison between LP20 and control **(A)**. Comparison between LP100 and control **(B)**. Comparison between LP500 and control **(C)**. CD, control diet; LP20, LP100 and LP500: 20, 100 and 500 mg of Feed LP20™ kg^-1^, respectively.

Moreover, DEGs from each diet comparison were used for enriched pathways analysis (**Figure 4**). LP20 diet (**Figure 4A**) induced a down-regulation of 2 terms related with oocyte meiosis and tight junction, but also up-regulated other 5 terms related with ECM-receptor interaction, focal adhesion, vascular smooth muscle contraction, regulation of actin cytoskeleton and TGF-beta signaling pathway. Furthermore, LP100 (**Figure 4B**) down-regulated 9 terms (e.g., inositol phosphate metabolism, oxidative phosphorylation, endocytosis and insulin signaling pathway) and up-regulated 13 terms (e.g., ECM-receptor interaction, cell adhesion molecules, phagosome, TGF-beta signaling pathway, MAPK signaling pathway and gap junction). Lastly, LP500 (**Figure 4C**) down-regulated 8 terms (e.g., ErbB signaling pathway, autophagy, endocytosis and TGF-beta signaling pathway), and up-regulated 15 terms (e.g., phagosome, cell adhesion molecules, gap and tight junction, PPAR signaling pathway and Calcium signaling pathway). Regarding the positive control diet, no modulated terms detected.

### 3.5 Protein detection

**Figure 5:**
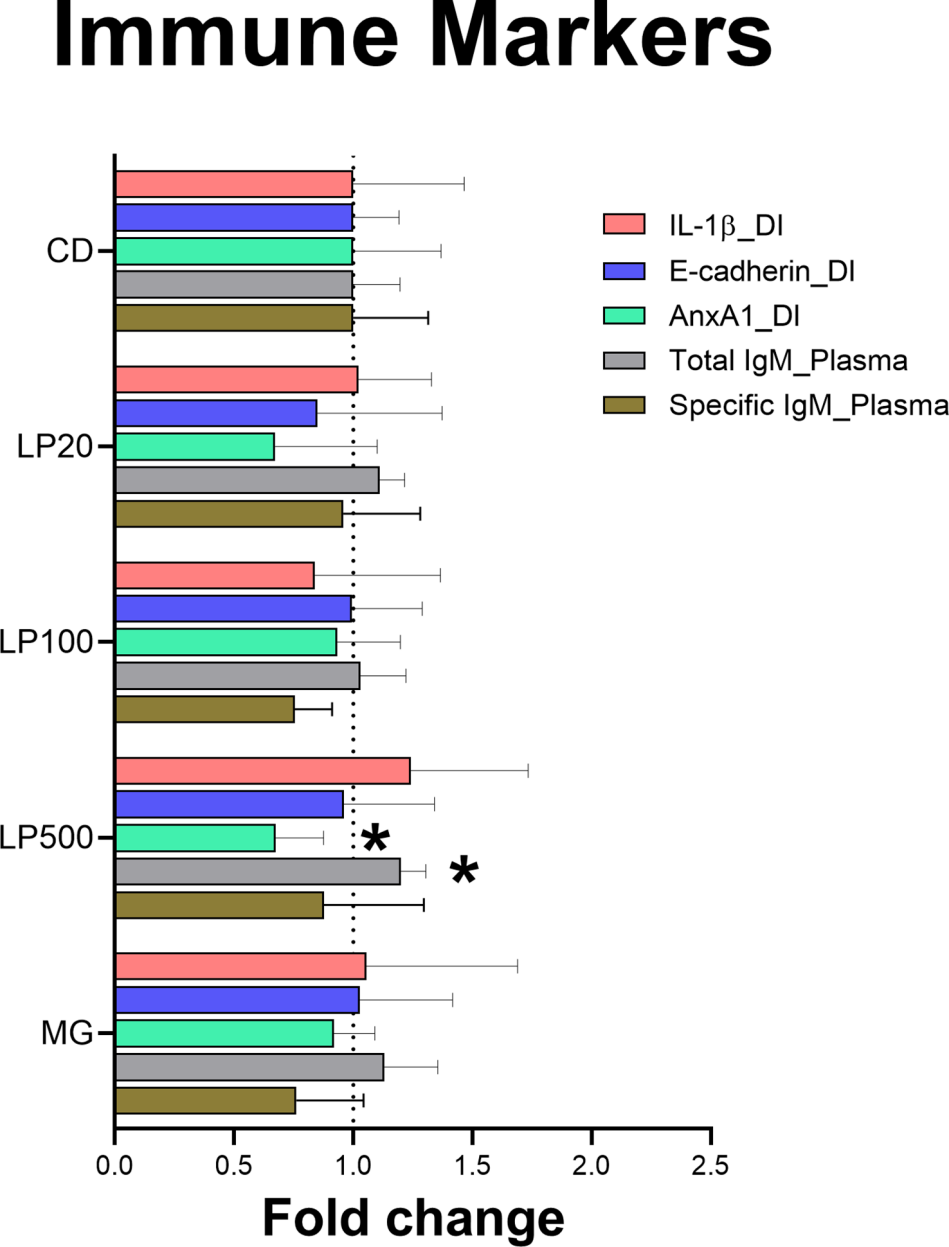
Protein detection of immune markers in distal intestine and plasma by indirect ELISA. Data is represented as fold change with error bars showing the standard deviation (n=9). Asterisk (*) denote level of significance (*p-value < 0.05). CD: control diet; LP20, LP100 and LP500: 20, 100 and 500 mg of Feed LP20™ kg^-1^, respectively; MG: positive control diet; IL-1β: Interleukin 1β; AnxA1: Annexin A1; IgM: immunoglobulin M; DI, distal intestine.

The results showed modulation of biomarkers related to immune response. The levels of Anxa1 were decreased in the distal intestine of fish fed LP500 (p-value = 0.0335). The same group also had a significant increase in the total IgM production in plasma (p-value = 0.0378) compared to the control diet (**Figure 5**). Protein levels of IL-1β and E-cad in distal intestine did not differ between experimental diet groups nor in positive control MG. Moreover, when measuring the production of specific IgM against HK L-137, there were no significant difference between groups.

## 4 Discussion

Immunonutrition is a promising cost-effective strategy to prevent considerable economic losses during fish production (6, 49). The effects of fish diseases, and subsequent mortality, are aggravated by several unavoidable stress factors such as handling, seawater transfer and pathogens (2). By providing functional feeds, immune response of fish can be enhanced, leading a more robust and healthy fish, able to cope better with such challenges (50). With this, stress, susceptibility to diseases and mortality can be mitigated, leading to improved animal welfare and a more sustainable aquaculture production.

Paraprobiotics, such HK L-137, are good candidates as functional ingredients. They can promote fish performance, modulate the intestinal microbiota and enhance the immune system (20, 21). However, when using a novel ingredient to a new species, and to respect animal welfare, *in vitro* trials are a desirable approach before animal experiments to predict inclusion levels and effects of the functional ingredients. At the *in vitro* level, after exposure to HK L-137, the lack of differences in TEER analysis confirmed similar integrity of the monolayer in both control and experimental groups due to proper development of tight-junctions between adjacent cells (30). When intestinal integrity is impaired, there is a reduction of digestive function and fish growth and fish becomes more vulnerable to bacterial infection (51, 52). Moreover, when Lucifer yellow was added apically, cells treated with HK L-137 reduced the passage of it, indicating a strengthened barrier function of the monolayer. Similar results were observed in the same model when used microbial or plant structural components (29) and with Caco-2 cells (colonic cells model of the intestinal epithelial barrier in humans), where *L. plantarum* strengthened the intestinal barrier function by promoting tight-junctions integrity (53). Later, the production of specific immune biomarkers, measured after 6 and 24 hours of HK L-137 exposure, showed a higher level of IL-1β in both time points, indicating an activation of the innate immune response. IL-1β has an important mediator role in fish as an earlier pro-inflammatory cytokine caused by an immunostimulant (54). Interestingly, after 24 hours treatment, there was a significant lower production of Anxa1, which supports the previous idea of activation of pro-inflammatory pathways, since Anxa1 has anti-inflammatory properties (55).

To characterize potential benefits to Atlantic salmon, an *in vivo* experiment was designed. After 61 days of feeding, no mortality was observed as well as no differences in fish performance between groups. Lack of differences in growth performance was also observed by Mohapatra et al. (56), where rohu (*Labeo rohita*) received different heat-inactivated probiotics, and in giant fresh water prawn (*Macrobrachium rosenbergii*) when fed HK L-137 (22). However, in previous studies where fish were fed HK L-137, improved growth performance was demonstrated (24–27), supporting the idea that the effects of paraprobiotics can be species-dependent (20). Histological analyses were performed in distal intestine. Sáenz de Rodrigáñez et al. (57) suggested that microvilli length of juvenile Senegalese sole (*Solea senegalensis*) was improved after administration of probiotics, for similar amount of time. However, in the current study, there were no signs of inflammation, and the villi height did not change among the groups. Together, these results suggest that HK L-137, at the current inclusion levels, is safe feed to Atlantic salmon without any deleterious effects.

When studying the intestinal microbiota of Atlantic salmon in this experiment, at the phylum level, there was an increase of the relative abundance of *Actinobacteriota* in the LP100 and LP500 groups due to the presence of *Mycobacterium* genus. Members of the genus *Mycobacterium* are found in wild and captive fish, and several species are considered pathogens (58). The route of infection is oral-intestinal pathway (59) and when infected, fish develop skin ulcers which lead to infection and increased mortality (60). However, none of these symptoms were observed in this experiment. Moreover, not all the fish reared in the same tank harbored this genus nor was it identified as top abundant genera in feed and water samples (**Figure S2**). Interestingly, fish fed MG also presented a relative higher abundance of genus *Mycobacterium* compared to the CD group. There was no indication of any consequences of the presence of this genus in the current study and no reports in Atlantic salmon were found. One can speculate that since these comparisons are considering relative abundance, in some fish groups, *Mycobacterium* had less competition for space or nutrients, which allowed its growth to become more abundant, and this was tolerated by the immune system. The genus *Stenotrophomonas* was significantly less abundant in the LP500 group (**Figure S1**). The role of this bacteria is debatable. Its protective role related to chitin degradation and anti-fungal activities has been related (61). However, their relative abundance was increased in healthy fish (62) and in fish after infection with an ectoparasite (63). In our study, the lower relative abundance of *Stenotrophomonas* could be a passive consequence of the increased relative abundance of another genus such as *Arenimonas* and *Mycobacterium*. *Ligilactobacillus* genus relative abundance was also significantly lower in the LP500 group (**Figure S1**). This genus belongs to the group of lactic acid producing bacteria (64) which are considered as beneficial due to their abilities to enhance digestive function, production of antimicrobial peptides and interaction with immune system (64). Nevertheless, other lactic acid bacteria were also identified: *Enterococcus*, *HT002*, *Lactobacillus*, *Ligilactobacillus*, *Limosilactobacillus*, *Peptostreptococcus*, *Streptococcus* and *Weissella*. The intestinal microbiota is redundant and dynamic regarding its function (65), which means that the same the function can be performed by several bacteria. In this case, the relative abundance of the sum of lactic acid bacteria did not differ between groups, not compromising the importance of lactic acid production and other related metabolites in the intestine. Furthermore, a dose-dependent trend of the relative abundance of *Proteobacteria* (currently known as *Pseudomonadota*) was also observed. At lower taxonomic levels, this tendency was more prominent, mostly caused by the genus *Arenimonas*. This genus is an environment related bacteria without described adverse effects on fish, however, due to the large individual variance, it was not significantly different. The remaining most abundant taxonomic groups did not differ between dietary groups.

Overall, the modulation of the microbiota in the digesta of distal intestine was minor, which was demonstrated by the lack of significant differences in diversity between the CD and the remaining groups. At the same time, such observation is not so surprising because we used paraprobiotic HK L-137 in small inclusion levels, ranging from 20, 100 and 500 mg of Feed LP20™kg^-1^ feed, (which means 5, 20 and 100 mg HK L-137 kg^-1^ feed). Because the bacterium was heat-killed, there was no obvious colonization, as also observed in previous studies (56). Therefore, there was no competition for space and nutrients with commensal bacteria. Instead, the observed effects of the paraprobiotic in the modulation of microbiota would need to act through other pathways, such as the epithelial cell metabolism and immune response, which in their turn could modulate the microbiota, as also suggested by Sun et al. (66).

Transcriptomic analysis of the distal intestine showed that the modulation of KEGG terms was related with the inclusion level and all groups had a higher number of up-regulated terms than down-regulated. All the terms up-regulated in LP20 were also up-regulated in the other inclusion levels (TGF-beta signaling pathway, reg. of actin cytoskeleton, vascular smooth muscle contraction, focal adhesion, and ECM-receptor interaction). Some of those terms are related with cell structure, cell adhesion, cell communication and proliferation and immune regulation (67–69), representing physiological and immunological benefits for the fish. The intestinal epithelium regulates the nutrient uptake and represents a crucial barrier function to the extrinsic environment, which when disrupted is commonly associated with an inflammatory response (70, 71). TGF-β is a cytokine with anti-inflammatory properties (72) and the distal intestine is part of the Gut-Associated Lymphoid Tissue (GALT), with a pivot importance in the innate immune response (73, 74). An up-regulation of terms related with TGF-beta signaling pathway, suggests an improved regulation of immune responses and prevention of an over-stimulation, contributing to the homeostasis in the distal intestine. Therefore, fish would benefit from an enhanced barrier function with a balanced immune response. In fish fed LP100 and LP500 diets, other terms related with immune response and barrier function were also up-regulated, such as phagosome term, MAPK signaling pathways, tight junctions, gap junctions and cell adhesion molecules. Phagosomes formation is part of innate immune system, involved in pathogen killing and antigen presentation (75), promoting to regulation of intestinal homeostasis. Moreover, MAPK signaling pathway is involved in the regulation of tight-junctions, which in turn, regulate paracellular transport (76). An appropriated regulation of tight junction, gap junctions and cell adherence, prevents then the undesirable passage of microorganisms or other antigens that can trigger an immune response, reinforcing the hypothesis that there is a positive dose dependent effect of Feed LP20™. The highest inclusion level (LP500) also led to an up-regulation of PPAR signaling pathway. PPARs are involve in the maintenance of metabolic homeostasis, however, PPARγ also has an important role in several immune cells (antigen-presenting myeloid dendritic cells and macrophages), regulating inflammation, antigen uptake and presentation, cell maturation and migration, and cytokine production (77). This indicate that HK L-137 has potential to enhance intestinal barrier function and immune response, which suggests positive effects to general fish health, as observed in previous studies with farmed fish (24–27). The up- and down-regulated KEGG terms in the same diet (cardiac muscle contraction in LP100, and MAPK signaling pathway and TGF-beta signaling pathway in LP500), could be due to the large number of genes these terms cover, thus while a group of these was up-regulated, another group might have been down-regulated within the biologic pathway.

At the protein level, higher inclusion levels of HK L-137 (LP500) showed a decreased production of Anxa1 in distal intestine (similar to what was observed at *in vitro* level using RTgutGC). In higher vertebrates, it has been described that a lower availability of Anxa1 could be involved in the polarization of T cells towards the Th2 effector subtype, which has then a critical role in the production of antibodies (78). We propose that the inclusion of LP500 would have similar effects on Atlantic salmon fed HK L-137, since we detected an increased production of total IgM in plasma, alike in Nile tilapia (79). Such modulation was not observed in fish fed MG diet. It could be suggested that the increased secretion of IgM in plasma could be directly against to the inclusion of a heat-killed bacterium in the feed. However, after analyzing the production of specific IgM against HK L-137, there were no significant differences. These results suggest that the IgM detected in plasma could be an increase in natural antibodies, which have crucial roles in pathogen elimination, B-cell survival and homeostasis (80). In addition, these humoral components could have a protective role by coordinating both innate and adaptive immunity (81).

Finally, the current study was performed on healthy fish, while future studies should combine HK L-137 and multi-stressor conditions or pathogen challenges to create an environment closer to commercial fish production.

## 5 Conclusion

In the current study, we investigated the potential of HK L-137 at *in vitro* level, by exposing a rainbow trout-derived intestinal epithelial cell line (RTgutGC) to HK L-137, and at *in vivo* level, with different inclusion levels of HK L-137 in pre-smolt feed. This approach was used to improve the experimental design when evaluating novel additives by an immunonutrition approach to promote animal health and welfare. In both levels, evidence of immunostimulant properties was observed from HK L-137 by modulating cytokines and effector molecules, suggesting its potential in functional feeds for salmonids without compromising fish performance and gut microbiota. This approach creates a baseline for future work involving HK L-137 and other pro- and paraprobiotics, both *in vitro* and *in vivo.*

## Data availability statement

RNA-seq raw data is available in Gene Expression Omnibus database (GEO-NCBI: GSE218341). The 16S rRNA gene sequencing raw data (fastaq files and metadata) are available in Sequence Read Archive, National Center for Biotechnology Information (SRA-NCBI: PRJNA907547). Other data and code for reproducing the results are available in the GitLab repository (https://gitlab.com/SergioRochaNMBU/hkl137_atlanticsalmon).

## Author contributions

MØ, PL, BM-L, LM and SR contributed to the conception of the study. PL conducted the *in vitro* trial. SR designed and formulated fish feed, conducted *in vivo* trial and sampling. SR analyzed histological images and 16S rRNA sequencing. BM-L analyzed indirect ELISA and transcriptomics. BM-L and SR performed bioinformatics, statistical analyses, and data visualization. SR wrote the first draft of the manuscript. All authors contributed to the article and approved the submitted version.

## Supplementary material

Supplementary Figure 1 Relative abundance of the top most abundant phyla and genera in distal intestine digesta samples. Data presented as mean abundance of each taxon within the same treatment (n=12). Asterisk (*) denote level of significance (*p-value < 0.05). CD: control diet; LP20, LP100 and LP500: 20, 100 and 500 mg of Feed LP20™ kg^-1^, respectively; MG: positive control diet.

Supplementary Figure 2 Relative abundance of the most abundant taxa in feed and tank water samples. **(A)** Relative abundance of top 10 most abundant phyla in feed samples. **(B)** Relative abundance of top 15 most abundant genus or lowest taxonomic group identified in feed samples. **(C)** Relative abundance of top 10 most abundant phyla in tank water samples. **(D)** Relative abundance of top 15 most abundant genus or lowest taxonomic group identified in tank water samples. **(A, B)** data presented as mean relative abundance from two technical replicates. **(C, D)** data presented as relative abundance from individual tanks (three tanks per treatment). CD: control diet; LP20, LP100 and LP500: 20, 100 and 500 mg of Feed LP20™ kg^-1^, respectively; MG: positive control diet.

Supplementary Figure 3 Antibody validation against Anxa1. (A) Affinity test against recombinant Anxa1 (rAnxa1) by indirect ELISA at 450 nm. **(B)** Three-dimensional model prediction by Phyre2. **(C)** The specificity to recognize Anxa1 was evaluated by Western blotting. The arrow indicates the band corresponding to the expected Anxa1 molecular weight (37 kDa).

Supplementary Figure 4 Measurement of single fold height. Data presented as mean with error bars representing the standard deviation (n=13-18) CD: control diet; LP20, LP100 and LP500: 20, 100 and 500 mg of Feed LP20™ kg^-1^, respectively; MG: positive control diet.
