# Supplementary figures and images for: From a cell model to a fish trial: Immunomodulatory effects of heat-killed Lactiplantibacillus plantarum as a functional ingredient in aquafeeds for salmonids
Source: Front Immunol. 2023 Mar 13;14:1125702. doi: 10.3389/fimmu.2023.1125702 (PMC10040762; doi:10.3389/fimmu.2023.1125702)

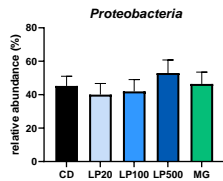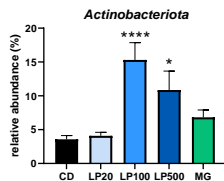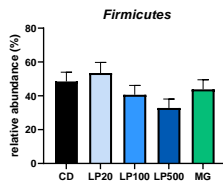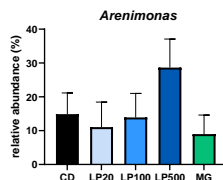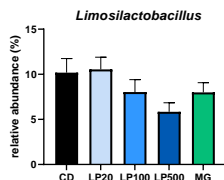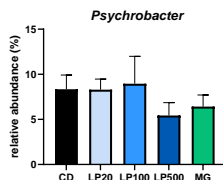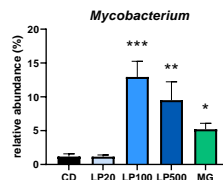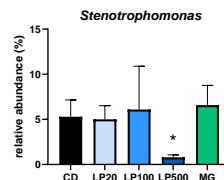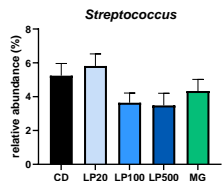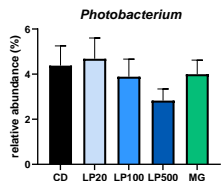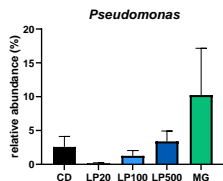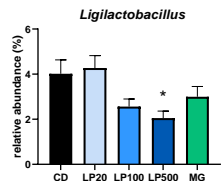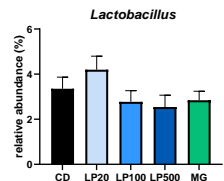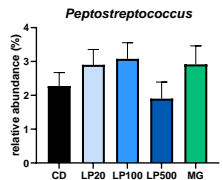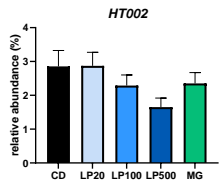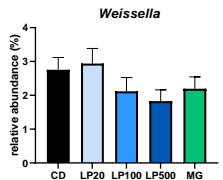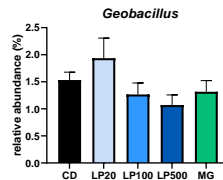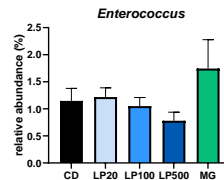

Supplement: Supplementary Figure 1 — Relative abundance of the top most abundant phyla and genera in distal intestine digesta samples. Data presented as mean abundance of each taxon within the same treatment (n=12). Asterisk (*) denote level of significance (*p-value < 0.05). CD: control diet; LP20, LP100 and LP500: 20, 100 and 500 mg of Feed LP20™ kg-1, respectively; MG: positive control diet. [file DataSheet_1.pdf]

A

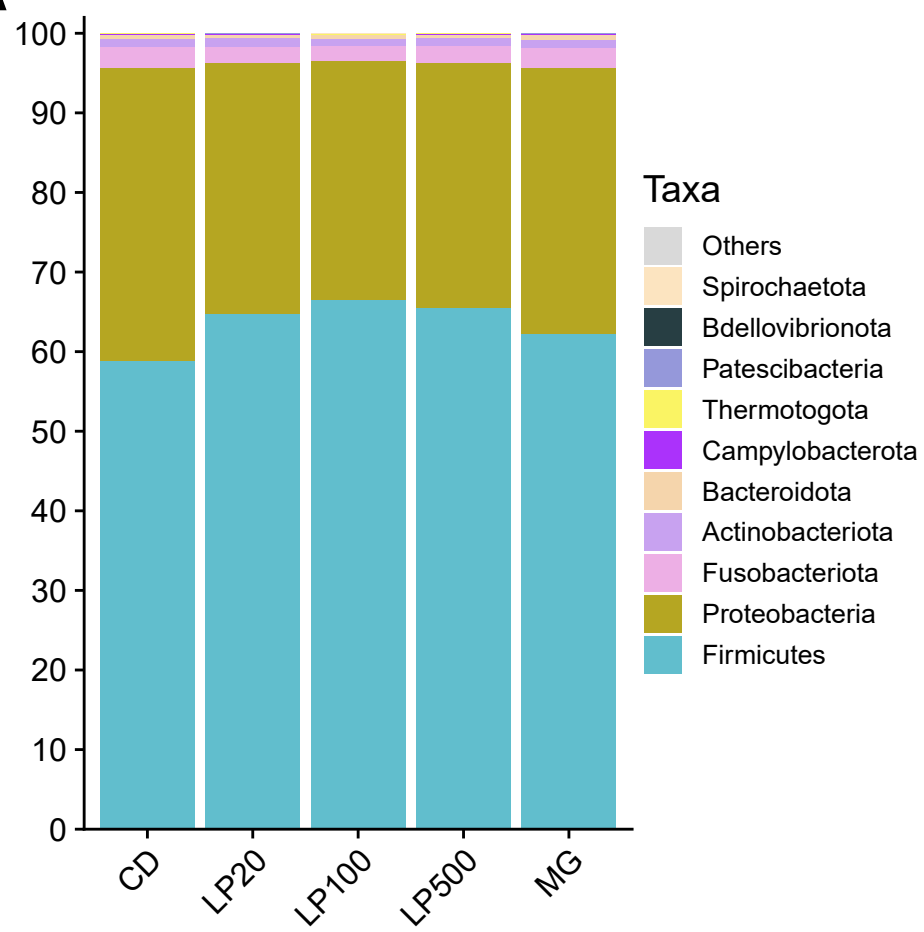

B

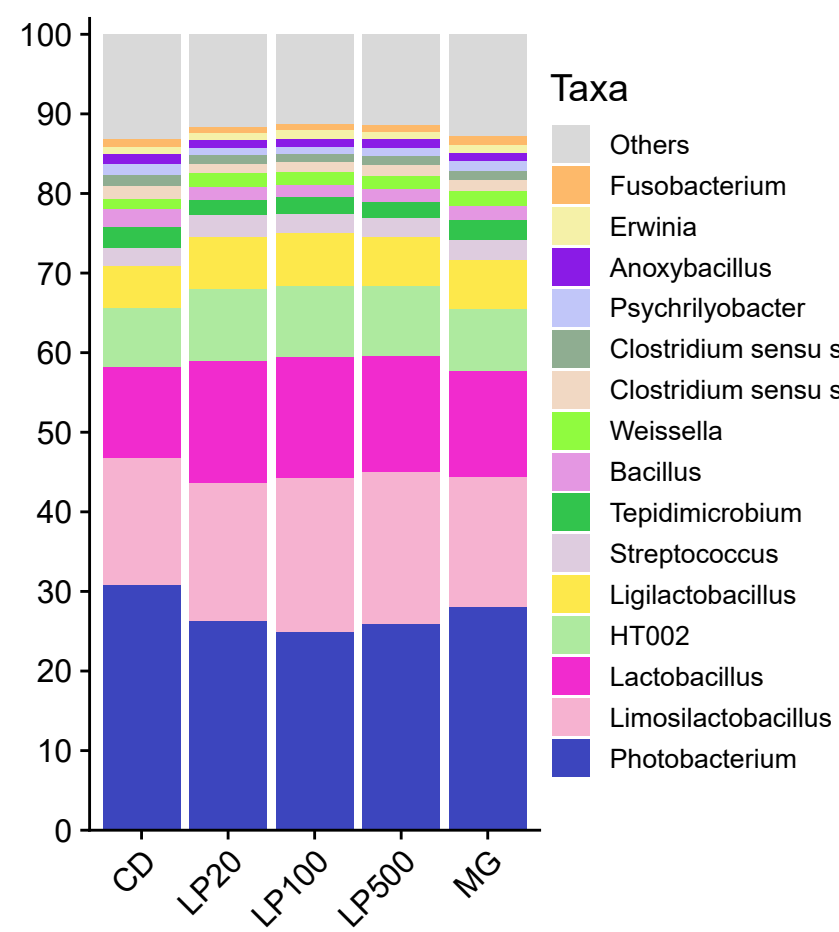

C

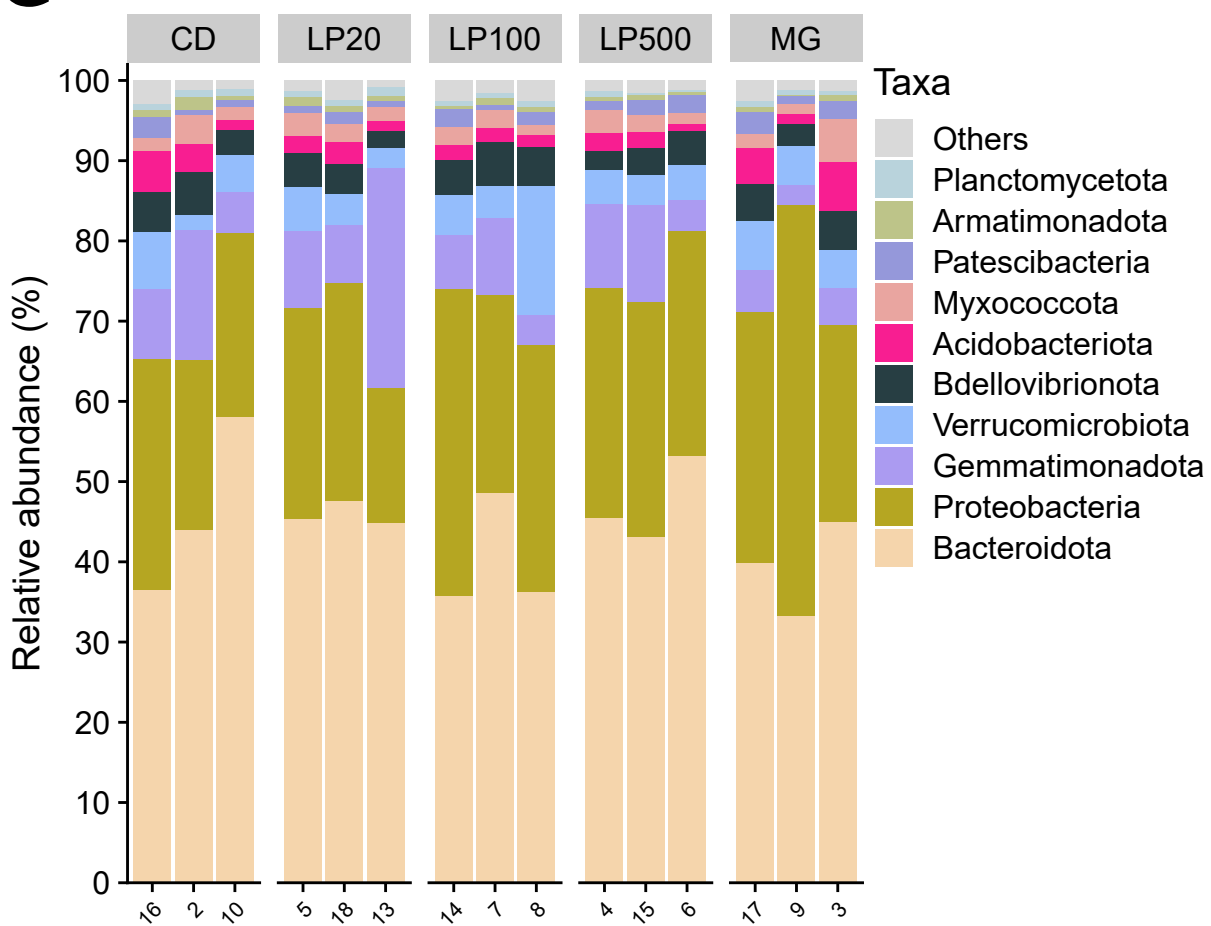

D

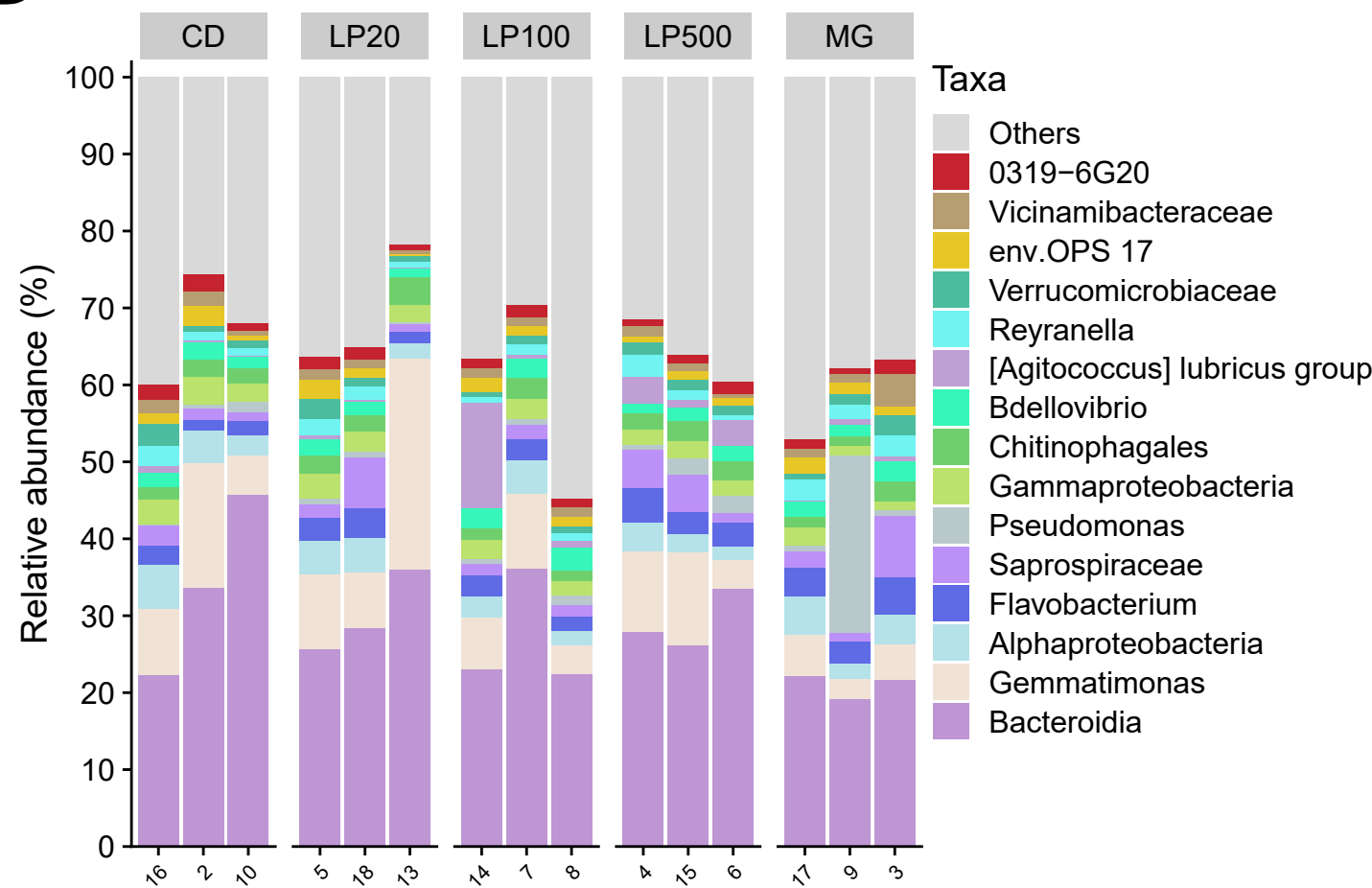

Supplement: Supplementary Figure 2 — Relative abundance of the most abundant taxa in feed and tank water samples. (A) Relative abundance of top 10 most abundant phyla in feed samples. (B) Relative abundance of top 15 most abundant genus or lowest taxonomic group identified in feed samples. (C) Relative abundance of top 10 most abundant phyla in tank water samples. (D) Relative abundance of top 15 most abundant genus or lowest taxonomic group identified in tank water samples. (A, B) data presented as mean relative abundance from two technical replicates. (C, D) data presented as relative abundance from individual tanks (three tanks per treatment). CD: control diet; LP20, LP100 and LP500: 20, 100 and 500 mg of Feed LP20™ kg-1, respectively; MG: positive control diet. [file DataSheet_2.pdf]

**A**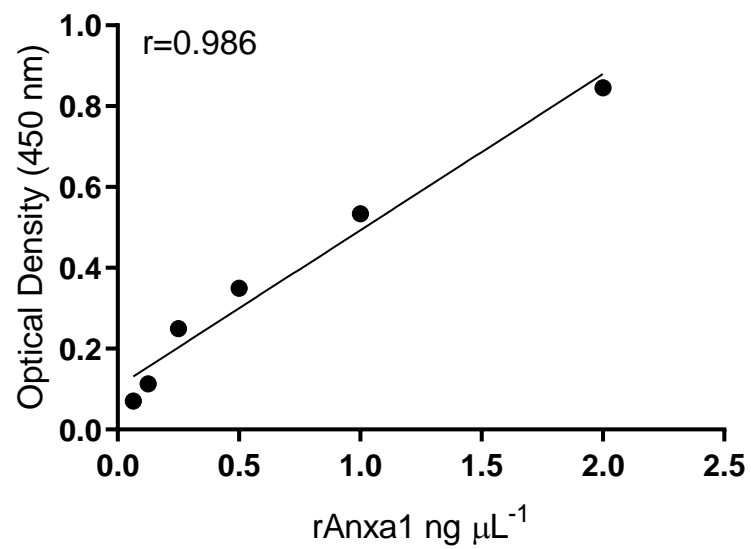**B**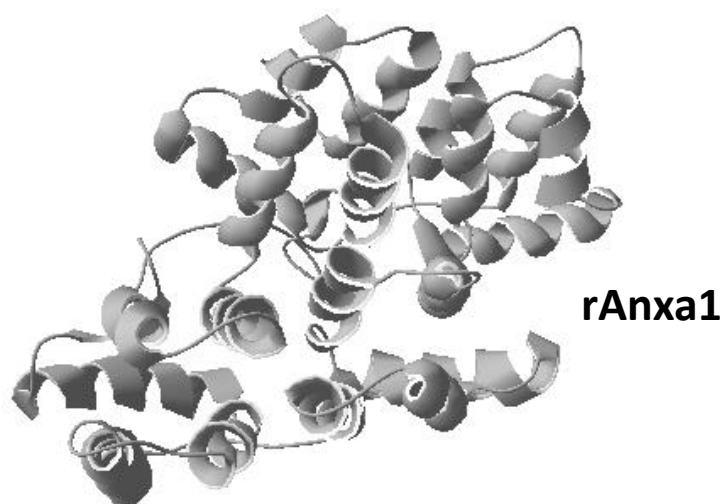**C**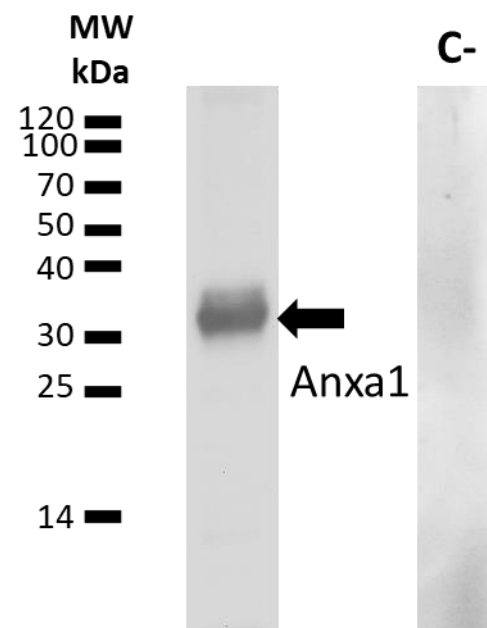

Supplement: Supplementary Figure 3 — Antibody validation against Anxa1. (A) Affinity test against recombinant Anxa1 (rAnxa1) by indirect ELISA at 450 nm. (B) Three-dimensional model prediction by Phyre2. (C) The specificity to recognize Anxa1 was evaluated by Western blotting. The arrow indicates the band corresponding to the expected Anxa1 molecular weight (37 kDa). [file DataSheet_3.pdf]

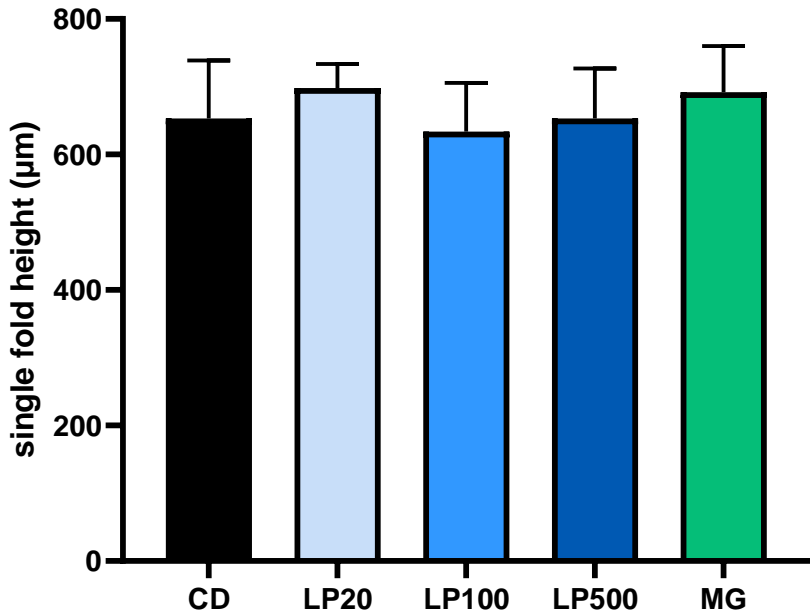

Supplement: Supplementary Figure 4 — Measurement of single fold height. Data presented as mean with error bars representing the standard deviation (n=13-18) CD: control diet; LP20, LP100 and LP500: 20, 100 and 500 mg of Feed LP20™ kg-1, respectively; MG: positive control diet. [file DataSheet_4.pdf]
